# Diacetyl control during brewery fermentation via adaptive laboratory engineering of the lager yeast *Saccharomyces pastorianus*

**DOI:** 10.1007/s10295-018-2087-4

**Published:** 2018-10-10

**Authors:** Brian Gibson, Virve Vidgren, Gopal Peddinti, Kristoffer Krogerus

**Affiliations:** 1 VTT Technical Research Centre of Finland Ltd, Tietotie 2, VTT, P.O. Box 1000, FI-02044 Espoo, Finland; 2 Department of Biotechnology and Chemical Technology, Aalto University, School of Chemical Technology, Kemistintie 1, Aalto, P.O. Box 16100, 00076 Espoo, Finland

**Keywords:** Diacetyl, α-Acetolactate, Chlorsulfuron, Beer, *Saccharomyces pastorianus*

## Abstract

Diacetyl contributes to the flavor profile of many fermented products. Its typical buttery flavor is considered as an off flavor in lager-style beers, and its removal has a major impact on time and energy expenditure in breweries. Here, we investigated the possibility of lowering beer diacetyl levels through evolutionary engineering of lager yeast for altered synthesis of α-acetolactate, the precursor of diacetyl. Cells were exposed repeatedly to a sub-lethal level of chlorsulfuron, which inhibits the acetohydroxy acid synthase responsible for α-acetolactate production. Initial screening of 7 adapted isolates showed a lower level of diacetyl during wort fermentation and no apparent negative influence on fermentation rate or alcohol yield. Pilot-scale fermentation was carried out with one isolate and results confirmed the positive effect of chlorsulfuron adaptation. Diacetyl levels were over 60% lower at the end of primary fermentation relative to the non-adapted lager yeast and no significant change in fermentation performance or volatile flavor profile was observed due to the adaptation. Whole-genome sequencing revealed a non-synonymous SNP in the *ILV2* gene of the adapted isolate. This mutation is known to confer general tolerance to sulfonylurea compounds, and is the most likely cause of the improved tolerance. Adaptive laboratory evolution appears to be a natural, simple and cost-effective strategy for diacetyl control in brewing.

**Electronic supplementary material:**

The online version of this article (10.1007/s10295-018-2087-4) contains supplementary material, which is available to authorized users.

## Introduction

Diacetyl (2,3-butanedione) is a vicinal diketone that imparts a distinct butter/butterscotch flavor and is an important component in the flavor profile of many foods [26]. In fermented beverages, diacetyl notes may be perceived positively or negatively depending on the product and style. In lager beers, which are characterized by fresh and clean flavor profiles, diacetyl is almost invariably considered as an off flavor. Consequently, the brewing process is carefully managed to minimize diacetyl levels. The long secondary fermentations carried out during lager brewing, for example, are primarily carried out to remove diacetyl from the system.

The precursor to diacetyl, α-acetolactate, is produced by yeast during fermentation. The compound is derived from cellular pyruvate through the action of the enzyme acetohydroxy acid synthase (Ilv2) and is a key intermediate in the valine synthesis pathway. α-Acetolactate is typically produced at levels exceeding metabolic demand and, as a result, some α-acetolactate typically diffuses across the cell membrane into the fermentation medium. Once released, α-acetolactate begins to be converted into diacetyl via a spontaneous non-enzymatic decarboxylation reaction. As this reaction occurs relatively slowly, the levels of pre-cursor are typically orders of magnitude higher than those of diacetyl [20]. Also, diacetyl, once formed, is rapidly taken up by yeast and reduced to less flavor-active compounds such as acetoin. Yeast is, therefore, involved indirectly in the production of diacetyl and involved directly in its removal. Any of the pre-cursor that remains in the beer after yeast removal is liable to be converted into diacetyl during beer storage and directly influence beer taste. It is, therefore, of critical importance that α-acetolactate (potential diacetyl) levels are kept to a minimum during production.

A number of strategies are used, or have been proposed, for diacetyl control [26]. Genetic modification of yeast for lowered acetohydroxy acid synthase activity, increased acetohydroxy acid reductoisomerase activity, increased diacetyl reductase activity [11, 30, 34, 40], or expression of foreign α-acetolactate decarboxylase [6], has been shown to be effective, but is not feasible due to current restrictions on the use of GM technology in food production. α-Acetolactate production varies with yeast strain, and strain selection is a simple strategy for diacetyl control [18]. However, strain changes are likely to impact on other beer quality parameters and this approach may not be suitable if flavor consistency is necessary. As α-acetolactate production is related to valine metabolism, changing amino acid composition of wort may influence diacetyl levels. Such changes are, however, also likely to influence beer character and are technically challenging due to the consistency of malt amino acid composition, and the technical challenge of supplementing wort with amino acids at appropriate levels [27]. Immobilized yeast reactors have been employed to rapidly remove diacetyl and obviate the need for secondary maturation [37]. Such systems involve rapid heating of beer after the primary fermentation to facilitate the conversion of the pre-cursor into diacetyl. The treated beer is then passed through a column of immobilized yeast where diacetyl reduction takes place. Though effective, this approach requires significant capital expenditure and runs the risk of heat-induced flavor change. Fermentation temperature and wort pH can influence the conversion of pre-cursor into diacetyl, but there is a limit to how far these parameters can be altered before an influence on beer quality is observed [28]. Direct provision of exogenous α-acetolactate decarboxylase can be effective [9], but represents an added cost in the process. An ideal strategy would result in lowered production of α-acetolactate by yeast, but would be achieved without changing the yeast strain, wort composition, fermentation conditions or brewery facilities. Such a strategy, if successful, would have a significant impact on the efficiency of the brewing process.

In an effort to meet the criteria listed above, an adaptive laboratory evolution approach was undertaken. Evolutionary engineering techniques have been used to alter numerous brewing and sake yeast phenotypes, including flavor profile [1, 15], sugar utilization [7] and stress tolerance [5, 14]. In the current study, the procedure involved exposure of a lager yeast population to chlorsulfuron, a sulfonylurea compound that specifically targets acetohydroxy acid synthase, the enzyme responsible for synthesis of α-acetolactate from pyruvate. Chlorsulfuron-exposed populations were screened for tolerant variants and these were further screened for their production of α-acetolactate during wort fermentation. The most promising isolate was used in a pilot brewery fermentation to assess the impact of any genetic changes on fermentation performance and flavor profile.

## Materials and methods

### Yeast and adaptation

The Frohberg-type lager yeast VTT A-63015, abbreviated here as A15, was used throughout. Yeast cells were exposed to chlorsulfuron at levels sufficient to impede growth, but remain sub-lethal. To establish effective concentrations, A15 cultures were inoculated into 50-ml YNB medium supplemented with all physiological amino acids, with the exception of valine and isoleucine (25 mg l^−1^ alanine, 30 mg l^−1^ arginine, 20 mg l^−1^ asparagine, 7.5 mg l^−1^ aspartic acid, 5 mg l^−1^ glutamine, 10 mg l^−1^ glutamic acid, 10 mg l^−1^ glycine, 10 mg l^−1^ histidine, 25 mg l^−1^ lysine, 10 mg l^−1^ methionine, 30 mg l^−1^ phenylalanine, 15 mg l^−1^ threonine and 10 mg l^−1^ tryptophan) and containing 4% (w/v) maltose in 100-ml Erlenmeyer shake flasks at a starting OD600 value of 0.1. Media were supplemented with chlorsulfuron by adding a stock solution (2 g l^−1^ dissolved in acetone) to achieve concentrations of 50–200 mg l^−1^. Acetone without chlorsulfuron was added to the control medium. Flasks were incubated at 18 °C with shaking (120 rpm) and growth was determined by regular OD600 measurement. The 100 mg l^−1^ treatment was found to reduce growth rate by 35% relative to a control (Fig. S1). Treatment did not result in cell death [viability was 99% after 120-h exposure as determined by propidium iodide staining using a Nucleocounter^®^ YC-100™ (ChemoMetec, Denmark)].

The procedure for strain adaptation was similar to that described by Ekberg et al. [14]. The yeast strain A15 was grown to stationary phase in Yeast Peptone medium (YP; 10 g of yeast extract and 20 g of peptone l^−1^) containing 40 g of maltose l^−1^. Yeast was harvested by centrifugation, washed with sterile water, suspended in 0.1 M sodium phosphate (pH 7.0) to 25 mg fresh yeast ml^−1^, and mutagenized with 20 µl ml^−1^ ethyl methanesulfonate (EMS) at room temperature (ca. 20 °C) for 60 min. The EMS reaction was quenched by adding 5 ml of sodium thiosulfate (50 g l^−1^). Mutagenized yeast cells were collected by centrifugation, washed twice with sodium thiosulfate (50 g l^−1^), and suspended in sterile saline (9 g NaCl l^−1^). The EMS exposure was mild, with less than a 5% drop in cell viability occurring as a result of treatment.

Mutagenized cells were inoculated into YNB medium supplemented with amino acids (as above), maltose (4%, w/v) and chlorsulfuron (100 mg l^−1^) to give a starting OD600 value of 0.1. Treatment involved three separate vessels which were incubated at 18 °C, with shaking (100 rpm) for 3.5 days. At this time, the OD600 values were measured and cells were transferred to fresh chlorsulfuron-containing medium, again at a starting OD of 0.1. The process was repeated until 30 transfers had been completed. In most cases, the yeast were grown in 1-ml medium in 2-ml cryovials. At transfer 15 and transfer 30, the culture volume was increased to 25 ml to obtain enough cells to prepare frozen stock cultures. These larger cultivations were carried out in 100-ml Erlenmeyer flasks, also at 18 °C and with shaking (100 rpm).

After transfers 15 and 30 (representing approx. 75 and 150 cell generations, respectively), samples of the cells were taken and stored at − 80 °C in 30% glycerol. These two cell populations, and the original EMS-treated cell population, were each transferred to three chlorsulfuron-supplemented plates at a viable cell density of 120 per plate, and incubated at 18 °C. The agar medium consisted of YNB with maltose (4%, w/v), the amino acid solution described above, and 500 mg l^−1^ chlorsulfuron. Agar was added at 1% (w/v). The higher concentration of chlorsulfuron was required as the 100 mg l^−1^ concentration used for adaptation was not effective in solid agar medium. The higher concentration allowed discrimination of adapted populations from non-adapted populations without being high enough to cause any loss of viability. Plates were incubated at 18 °C and colony appearance was monitored over a 10-day period.

Seven early-appearing colonies were isolated for further analysis. These were colonies that appeared 4 days after cells were spread on chlorsulfuron-containing plates and included 4 isolates from transfer 15 (Isolates 1, 6, 7, and 8), and three isolates from transfer 30 (Isolates 15, 16, and 20). 30% glycerol stock cultures were prepared and stored frozen at − 80 °C until required.

### Wort preparation

To prepare wort, 26 kg of hammer-milled pilsner malt was used to produce approx. 100 l of 15°P wort. Water was added at a ratio of 3:1. The mash was supplemented with 52 ml strong lactic acid, 30 g CaCl·2H_2_O, 10 g CaSO_4_·2H_2_O and 53 mg ZnSO_4_·7H_2_O. Mash profile was as follows: 48 °C, 30 min; 63 °C, 30 min; 72 °C, 30 min and 78 °C, 10 min. A Meura filter was used for wort separation. Wort was boiled for 60 min with Magnum hops (15% alpha acid). Hot trub separation was achieved by whirlpool.

### Fermentations

Initial screening fermentations were conducted with A15 and the 7 chlorsulfuron-adapted isolates. Frozen stock cultures were used to start 100-ml cultivations in YP medium containing 4% maltose. After 48 h’ growth at 20 °C with shaking (120 rpm), OD600 values were calculated and the isolates were inoculated into 500 ml 15°P brewer’s wort in 1-l Erlenmeyer flasks at a starting OD600 value of 0.1. Flasks were incubated at 18 °C with shaking (80 rpm). After 5 days, the cultures were transferred to 0 °C and yeast were allowed to sediment. The fermented wort was decanted to give a 20% yeast slurry (200 g fresh yeast l^−1^). The yeast slurry was added to 1.5 l of 15°P, oxygenated (10 ppm), all-malt wort in sterile, stainless-steel ‘tall tubes’ to give an inoculation rate of 5 g l^−1^. These fermentation vessels had an internal volume of 2 l, internal diameter of 6 cm and height of 100 cm [39]. Fermentations were carried out at 15 °C and samples for vicinal diketone (VDK) analysis were taken 3 days after inoculation when concentrations were expected to be close to peak levels.

For pilot-scale fermentations, yeasts were propagated in YP medium containing 4% maltose as above, and inoculated into a generation 0 ‘G0’ fermentation of 30 l, 15°P all-malt wort in 50-l-volume cylindroconical fermentation vessels. Fermentations were conducted at 15 °C and yeast were harvested from the base of the fermenter after 10 days. A 20% slurry was prepared by decanting as before. This slurry was used for inoculation of the experimental ‘G1’ fermentations, which consisted of 30 l of 15°P wort aerated at 10 ppm dissolved oxygen in a 50-l-volume cylindroconical vessel. Yeast were pitched at a rate of 5 g fresh yeast l^−1^ and fermentations were conducted at 15 °C. The G1 fermentations were monitored as the vast majority of industrial brewery fermentations are fermented with recycled (re-pitched) yeast rather than freshly propagated yeast.

Wort samples were drawn aseptically from the fermentation vessels on a regular basis, and placed directly on ice, after which the yeast was separated from the fermenting wort by centrifugation (9000×*g*, 10 min, 1 °C). Yeast viability was measured from the yeast that was collected at the end of the fermentations by propidium iodide staining using a Nucleocounter^®^ YC-100™ (ChemoMetec, Denmark).

### Wort and beer analyses

The density, alcohol concentration and pH of the samples were determined from the centrifuged and degassed fermentation samples using an AntonPaar Density Meter DMA 5000 M with Alcolyzer Beer ME and pH ME modules (AntonPaar GmbH, Austria). The yeast pellet of the samples was washed with deionized H_2_O and centrifuged again to determine the mass of yeast in suspension.

Total diacetyl and 2,3 pentanedione (combined free and acetohydroxy acid form) in the centrifuged fermentation samples was measured according to Analytica-EBC method 9.10 [13]. Samples were heated to 60 °C and kept at this temperature for 90 min in a headspace auto-sampling unit (Headspace Autosampler 7000 HT, Tekmar-Dohrmann, USA). Heating to 60 °C results in the conversion of acetohydroxy acids into VDKs. The samples were then analysed by headspace gas chromatography (HP 6890 Series GC System, Hewlett-Packard, USA; HP-5 50 m × 320 µm × 1.05 µm column, Agilent, USA) with 2,3-hexanedione as an internal standard. The ‘total diacetyl’ results are a good indication of α-acetolactate levels due to the fact that free diacetyl is rapidly reduced by yeast, and therefore, only detectable at low concentrations in wort [20].

Aroma compounds (higher alcohols and esters) were measured by headspace gas chromatography with flame ionization detector (HS-GC-FID) analysis. Filtered (0.45 μm) samples were first incubated for 30 min at 60 °C, and then 1 ml of gas phase was injected (split mode; 225 °C; split flow of 30 ml min^−1^) into a gas chromatograph equipped with a FID detector and headspace autosampler (Agilent 7890 Series; Palo Alto, CA, USA). The carrier gas was helium (constant flow of 1.4 ml min^−1^). The temperature profile was 50 °C for 3 min, raised to 100 °C by 10 °C min^−1^, to 140 °C by 5 °C min^−1^ and to 260 °C by 15 °C min^−1^, followed by isothermal conditions for 1 min. Compound identification was done by comparing authentic standards and quantified with standard curves. 1-Butanol was used as internal standard.

### Genome analysis

A15 and an adapted variant of A15 (Isolate 8) were sequenced by Biomedicum Genomics (Helsinki, Finland). In brief, an Illumina NexteraXT pair-end 150 bp library was prepared for each yeast and sequencing was carried out with a NextSeq 500 instrument. Pair-end reads from the NextSeq 500 sequencing were quality analysed with FastQC [3], and trimmed and filtered with Skewer [25]. Reads were aligned to a concatenated reference sequence of *S. cerevisiae* A62 [29] and *S. eubayanus* FM1318 [4] using BWA-MEM [33]. Alignments were filtered to a minimum MAPQ of 50 with SAMtools [32] and the quality of alignments was assessed with QualiMap [16]. The median coverage over 10,000 bp windows was calculated with BEDTools [38] and visualized with R (http://www.r-project.org/). Variant analysis was performed on the aligned reads using FreeBayes [17]. Variants with a quality score less than 20 and read depth less than 30 were discarded. Variants were annotated with SnpEff [10]. Copy number variations were estimated with CNVKit [41]. Genome data have been deposited at NCBI as BioProject 475670 (http://www.ncbi.nlm.nih.gov/bioproject/PRJNA475670).

### Confirmation of the ILV2 missense mutation by Sanger sequencing

The heterozygous 574 C > T missense mutation in the *S. cerevisiae* allele of *ILV2* that was observed in the adapted variant of A15 (Isolate 8) was confirmed by Sanger sequencing. First, a fragment consisting of the first 684 bp of *S. cerevisiae ILV2* was amplified with PCR using the following primers: ScILV2_1-22_fw: 5′-ATGATCAGACAATCTACGCTAA-3′, and ScILV2_665-684_rv: 5′-CAATGGCAATTCTTCCACGG-3′. The amplicon was cleaned using the QIAquick PCR purification kit of Qiagen (Hilden, Germany), and sequenced at Seqlab-Microsynth (Goettingen, Germany). The chromatograms were aligned and visualized in Geneious 10.0.9 (Biomatters).

## Results

**Fig. 1:**
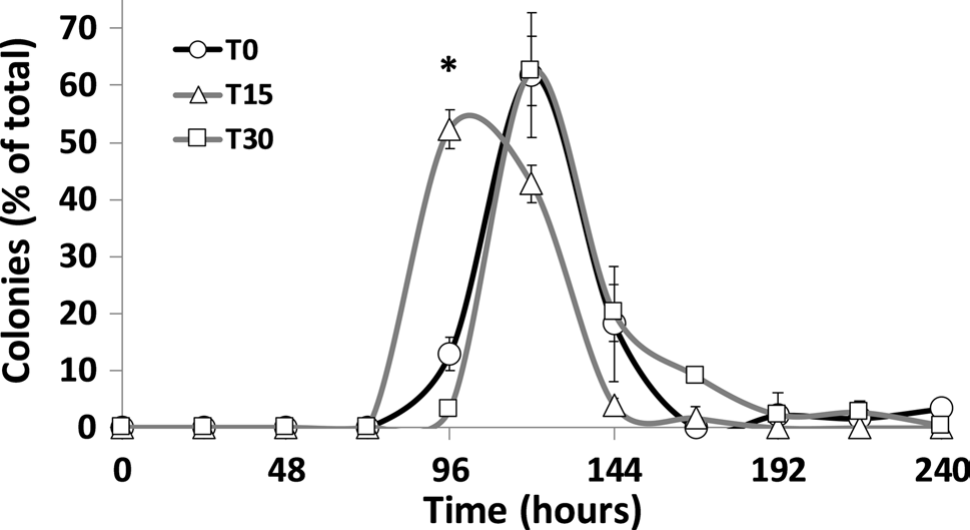
Emergence of visible colonies from control and chlorsulfuron-exposed populations of the A15 lager yeast after spreading cells on agar plates supplemented with 500 mg l^−1^ chlorsulfuron. Populations included a control population after EMS treatment, but not previously exposed to chlorsulfuron (T0), and populations exposed to 15 or 30 serial transfers to liquid media containing 100 mg l^−1^ chlorsulfuron, T15 and T30, respectively. The number of new colonies emerging each day is expressed as a percentage of the total number of visible colonies after 10 days. Results are averages from three replicate agar plates each producing at least 120 visible colonies on day 10. Asterisk denotes significant difference (*p *< 0.01) at this time point as determined by unpaired, two-tailed Student’s *t* test

Repeated chlorsulfuron exposure resulted in improved tolerance. Cell populations exposed to 100 mg l^−1^ chlorsulfuron formed visible colonies earlier than control populations when transferred to agar plates containing the compound (Fig. 1). 52% of colonies were visible after 96 h, compared to only 13% for the control population (*p *< 0.01 as determined by unpaired two-tailed Student’s *t* test). This was, however, only observed with the cells that had been exposed to 15 consecutive transfers to the media. The colony-forming potential appeared to decrease with the populations that were passaged 30 times through the same media. Nevertheless, some early forming colonies did appear at 96 h from the T30 population and these were included in a subsequent test for diacetyl formation. All populations produced the approx. 120 colonies expected per plate, i.e., only time of emergence differed between populations and not cell viability due to the sub-lethal concentration of chlorsulfuron used.

**Fig. 2:**
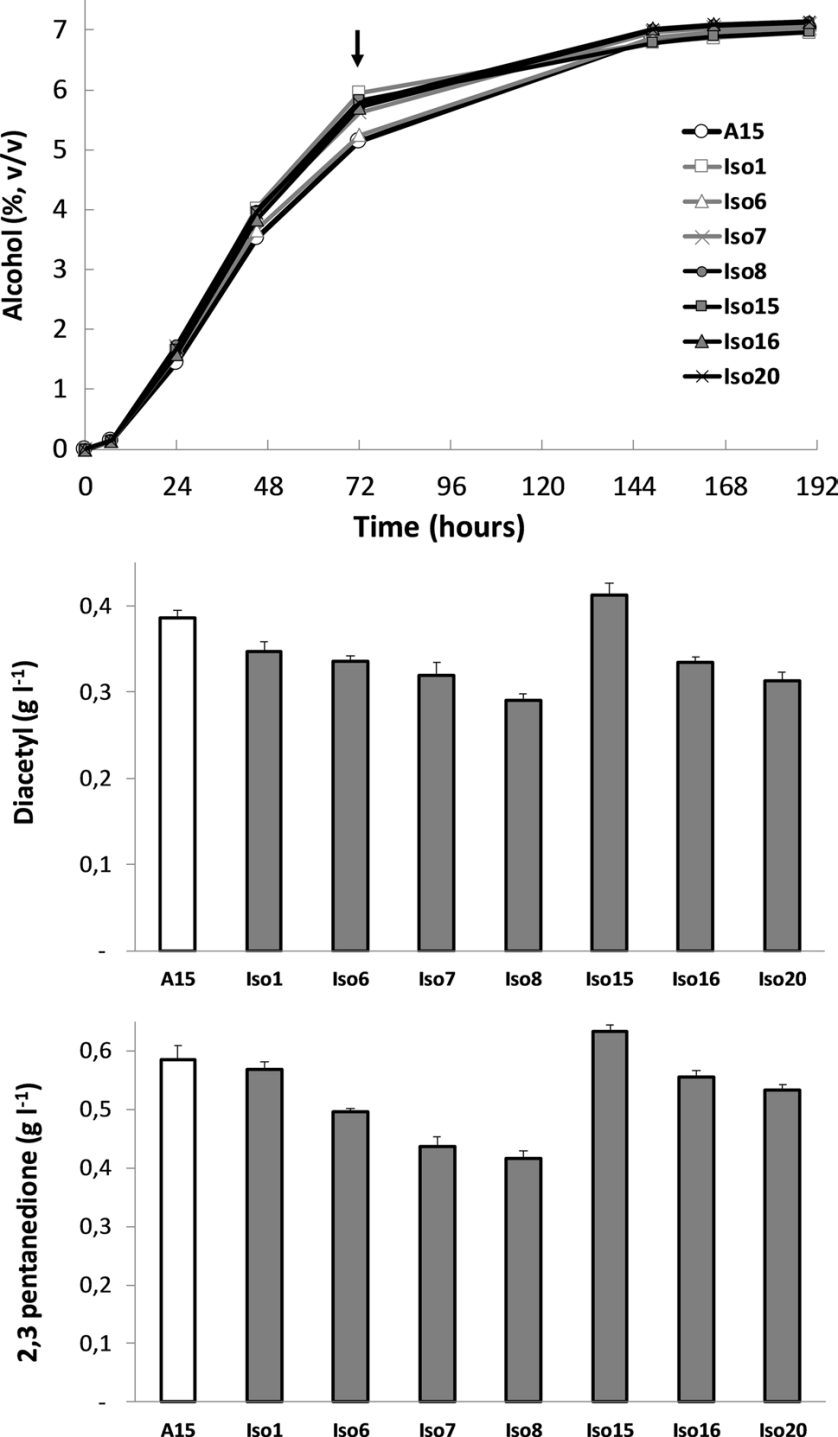
Preliminary assessment of alcohol evolution and VDK level during fermentation of all-malt 15°P wort with the lager yeast A15 and chlorsulfuron-adapted isolates of A15. Concentration of the vicinal diketones diacetyl and pentanedione were measured in samples taken 72 h following inoculation, as indicated in the upper panel. Values are from single fermentations. VDK data are means of three technical replicates and error bars indicate standard deviation

An initial screening for fermentation performance and VDK production involved 8 strains. The A15 lager strain and the 7 selected isolates were used to inoculate 2 l of 15°P all-malt wort at a pitching rate of 5 g l^−1^. Alcohol production was largely unaffected in the strains tested (Fig. 2). However, at the 72-h sampling time, a difference in alcohol content was observed, with most adapted strains having a higher value (5.6–5.9% alcohol, v/v) than the reference strain A15 (5.1%). The exception was Isolate 6, where at 72 h, the alcohol content was 5.2% and the fermentation rate was similar to that of A15 throughout the fermentation. VDK levels in worts fermented with the chlorsulfuron-adapted isolates were generally lower at 72 h, the time when peak levels are typically observed [28]. These differences were not apparently related to fermentation rate, as one of the faster fermenting strains (Isolate 15) had similar diacetyl and 2,3 pentanedione levels compared to A15. Likewise, the strain with an apparently identical fermentation profile to the reference strain had a lower VDK value. Diacetyl level was reduced by as much as 10%, e.g. in the case of Isolate 8. The extent of reduction was similar, though not identical, for 2,3 pentanedione. Again, Isolate 8 was the one producing the lowest concentration at 72 h, with, in this case, a 17% reduction relative to the reference strain. The isolates did not show any clear difference in growth, flocculation or wort pH during fermentations (data not shown). Based on this initial screening, Isolate 8 was chosen for confirmation of the lower production of α-acetolactate, and for a more thorough analysis of fermentation performance and beer attributes relative to the original strain.

**Fig. 3:**
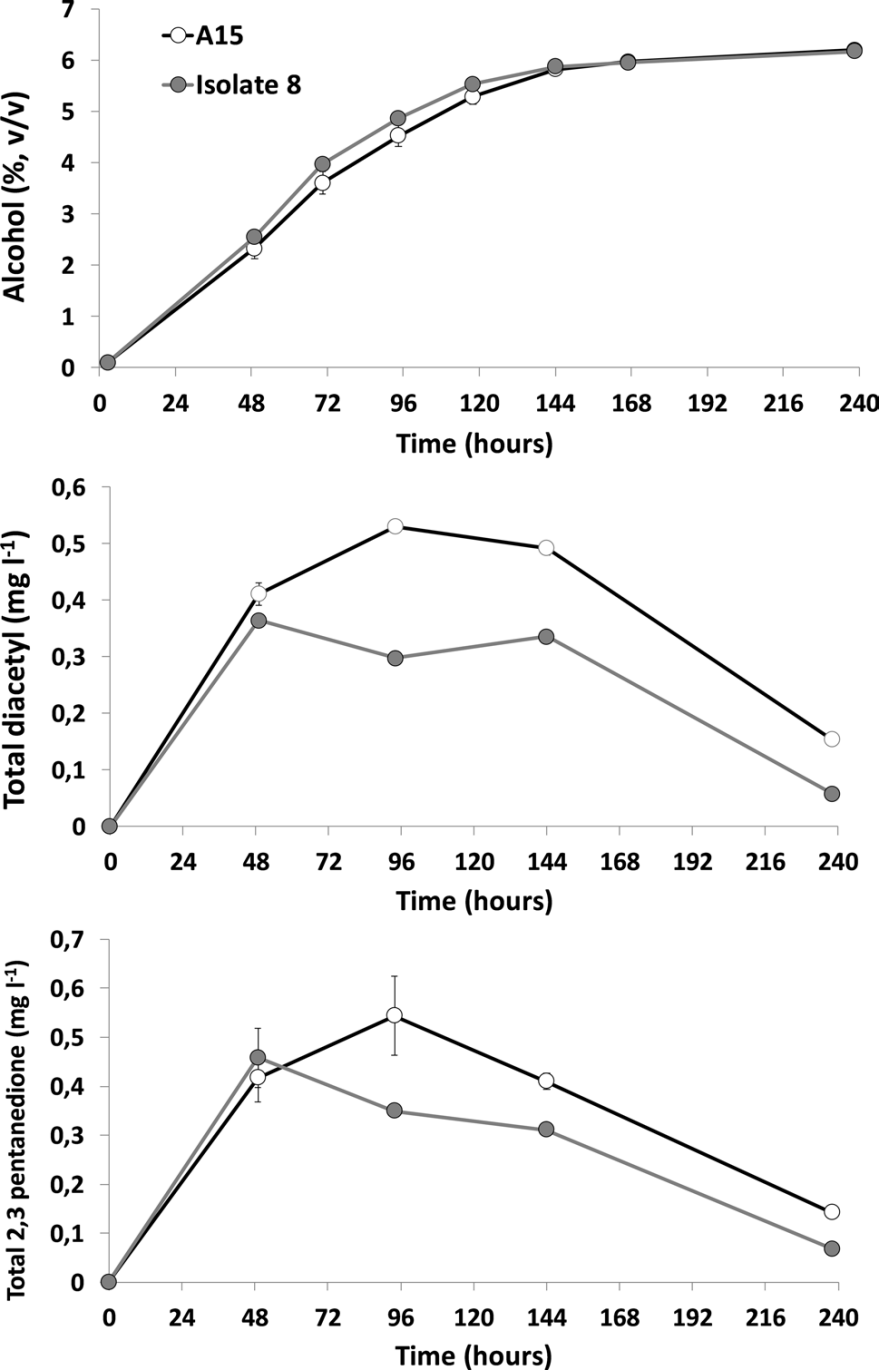
Alcohol content, total diacetyl and total 2,3 pentanedione concentrations during fermentation of 15°P all-malt wort at 30-l scale with the lager strain A15 (open symbols) and an A15-derived, chlorsulfuron-adapted variant (Isolate 8; closed symbols). Values are means of two replicates and error bars where visible indicate the range about the mean

Performance of the reference strain and Isolate 8 during 30-l-scale fermentations was consistent with previous results. A slightly improved fermentation rate was observed with Isolate 8 after 48 h, consistent with a greater uptake of maltose from the wort. After 5 days, differences were no longer apparent and alcohol yield was 6.2% for both yeast. Yeast mass in suspension and pH were generally the same for both yeast strains and there was no indication that the adapted yeast had suffered from any ‘crippling’ changes during the adaptation (Fig. S2). The greatest change was observed for total VDK levels. At 4 days after inoculation, when VDK levels are expected to be close to their peak values, there was a markedly lower level of both diacetyl and 2,3 pentanedione (Fig. 3). In the former case, a 45% reduction relative to the reference is observed at 4 days (*p *< 0.01 as determined by unpaired two-tailed Student’s *t* test). The lower production is apparent still at the end of fermentation, where the diacetyl levels are over 60% lower relative to the reference (*p* < 0.01 as determined by unpaired two-tailed Student’s *t* test). A similar situation was observed for 2,3 pentanedione with levels being 36% and 53% lower at 4 days and 10 days, respectively, in Isolate 8 fermentations relative to A15 fermentations. The similar trends seen for both compounds suggest that a common mechanism is responsible for the changes observed.

Higher alcohol production during fermentation was not greatly affected by the strain used (Fig. S3). Concentration of phenylethanol at the end of the Isolate 8 fermentation was higher at 19 mg l^−1^ compared to A15 at 16.5 mg l^−1^. Both values are below the recognized flavor threshold for this compound [36] and the difference is not expected to influence flavor perception. The similar concentrations of 2-methylpropanol and 2-methylbutanol in both worts suggest that the uptake of the precursors valine and isoleucine was not influenced by chlorsulfuron adaptation. Altered amino acid uptake is, therefore, unlikely to be the reason for the observed differences in diacetyl and 2,3 pentanedione in wort. Concentrations of esters in the respective fermentations did not differ greatly (Fig. S4). This was true for both ethyl esters and acetate esters. The one exception was 3-methylbutyl acetate, where the concentration was somewhat lower in Isolate 8, relative to A15 (1.0 mg l^−1^ vs 1.2 mg l^−1^). Both values are below the flavor threshold for this compound and the difference noted is unlikely to influence flavor perception.

**Fig. 4:**
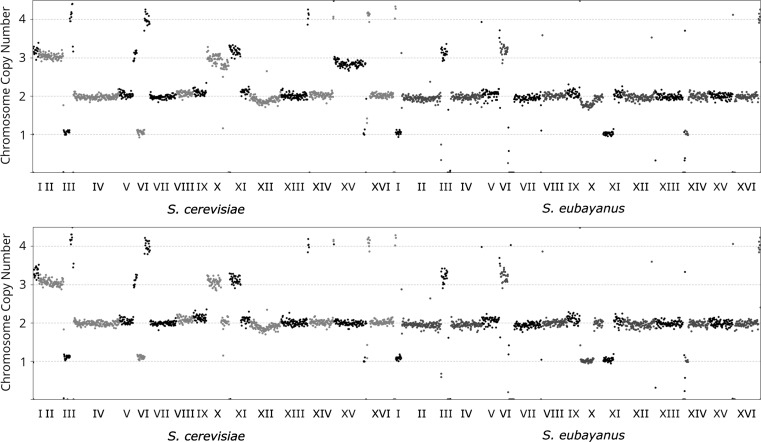
Genome coverage of the A15 lager strain (above) and A15 variant Isolate 8 (below) showing the contribution of individual chromosomes to the genome and the changes occurring after adaptation for lowered diacetyl production

Due to the clear differences in diacetyl production of A15 and the variant strain, it was considered useful to compare the genome sequences of both strains in an attempt to identify the underlying genetic changes responsible for the observed differences. Genome coverage data indicated the loss of a number of chromosomes in the variant strain. These included a whole copy of the *S. cerevisiae*-derived chromosome XV and one copy of a chimeric chromosome X, consisting of the left arm of the *S. eubayanus*-derived and the right arm of the *S. cerevisiae*-derived chromosome X [21] (Fig. 4). Altogether, the chromosome losses led to the loss of 1075 individual gene copies. However, complete loss of all copies of a particular gene was extremely rare, only occurring for the ligase genes *AIM22* and *RTT101*. Of the genes that are known to directly influence α-acetolactate metabolism, the only one that was influenced by gene loss was *ILV3*, encoding a dihydroxyacid dehydratase involved in valine metabolism. It is unlikely that this particular change would contribute to lowered diacetyl levels in beer as reduced activity of this enzyme would be expected to increase diacetyl level. It is rather likely that its loss here is incidental.

SNP analysis revealed that a number of genes in the variant strain contained mutations not seen in the original strain. A total of 130 non-synonymous mutations unique to Isolate 8 were detected (Table 1 and Table S1), and these include insertions, deletions and frameshift mutations. Of particular note is the missense mutation occurring in a *S. cerevisiae* copy of the gene *ILV2*. This mutation (confirmed with Sanger sequencing; Fig. S5 in the supplementary material), position 574 C > T, resulted in a change in amino acid sequence (proline to serine at position 192) and may have influenced the function of the gene. This conjecture is supported by the fact that the same mutation has been observed previously in a yeast culture exposed to toxic levels of the acetohydroxyacid synthase inhibitor sulfometuron methyl [46]. The mutation in that case led to resistance to the inhibitor and reduced sensitivity to valine. A similar resistance to the enzyme inhibitor may be occurring in Isolate 8, despite the fact that a different inhibitor (chlorsulfuron) was utilized in the adaptation. As *ILV2* has a key role in the production of α-acetolactate, it is likely that this mutation is responsible for the altered diacetyl levels seen in the variant beers. The genes listed in Table 1 include 9 nonsense mutations and 121 missense mutations. The former group contains no genes that are directly relevant to the acetohydroxy acid production, while the latter group includes 7 genes (*GLT1*, *LYS4*, *ARO10*, *MET13*, *MES1*, *ILV2*, *ARG8*) belonging to the gene ontology term ‘cellular amino acid metabolic process’ (GOID6520) and these mutations (all heterozygous) may have had some impact on general amino acid metabolism that may have influenced production of acetohydroxy acid. The *ARO10* gene may be of particular importance as the phenylpyruvate decarboxylase encoded is directly involved in amino acid metabolism via decarboxylation of α-keto acids to aldehydes [43].

**Table 1:** Genes affected by missense or nonsense mutations detected in the low-diacetyl variant strain Isolate 8 relative to the original A15 lager strain

Sub-genome	Missense mutation	Nonsense mutation
*S. cerevisiae*	*AFG2*, *AIM33*, *AKR2*, *CCC2*, *CDC14, COX10, DUF1, DYN1, EIS1, ERO1, GEA2, GRE3, GTT2, HTA1,* ***ILV2****, IML1, KNS1, KRE5, MES1, MPS1, MTC5, NEW1, NOT3, OYE2, PET111, PET127, PEX13, PEX6, PGD1, POB3, PRP2, PRP22, PTC2, PTC5, RAD54, RIC1, ROM1, RPH1, RPS7B, RPT2, RTN2, SCH9, SEC24, SNF2, SSL2, STI1, TAH18, TES1, THI22, THI72, TMA108, TOF2, UBP12, URB1, USA1, WHI4, VHT1, VTA1, YLR287C, YNR021W, YPL039W*	*HRD3, MEF2, POL1, SPP2, TEA1*
***S. eubayanus***	*ALE1, ARG8,* ***ARO10****, BEM1, BEM2, CHO2, COP1, COQ6, DOP1, DYS1, EAR1, FAP7, FPK1, GLT1, HAP1, KSP1, LYS4, MET13, MMS4, MPM1, MTF1, NHA1, NTE1, PEX10, PMD1, POL4, RAD5, RAD7, RIP1, RPN1, RPN7, SAS3, SKG6, SMK1, SPB1, SPT6, SRC1, SSL2, STB5, TFC4, TIF4632, TIP41, UBP1, WHI2, VHS2, VPS45, YBL036C, YGR026W, YGR067C, YHR127W, YLR146W*-*A, YMR209C, YMR210W, YPS6, YTA6, ZRC1*	*MTW1, NCE103, NSE5, TRE1*

See Table S1 for details

## Discussion

Chlorsulfuron was shown to be an effective selection agent for diacetyl control. Peak levels of diacetyl were reduced by 45%, and green beer levels by over 60%, without any notable impact on fermentation performance or beer quality. The adaptive laboratory evolution approach has generally been applied to brewing yeast in an effort to improve stress tolerance or fermentation efficiency [5, 7, 14, 23]. These examples have involved alteration of phenotypes with a direct and distinct adaptive advantage with regard to survival or growth. The approach used here is indirect, in the sense that a somewhat higher or lower production by yeast cells of the diacetyl precursor is not expected to have a direct impact on the survival or fermentation performance of the strain. Similar approaches have been used, mainly in the Japanese sake industry, to modify the production by yeasts of other specific flavor compounds. These have included increased 3-methylbutyl acetate (banana/pear aroma) by lager yeast after exposure to 5,5,5-trifluoro DL-leucine [31] and by sake yeast after exposure to isoamyl monofluoroacetate [44], isoamyl monochloroacetate [45] and 1-farnesylpyridinium [22]. A fivefold increase in ethyl caproate (apple aroma) in sake yeast was observed by Ichikawa et al. after adaptation to cerulenin [24]. The rose aroma phenylethyl acetate was also increased in lager yeast through exposure to fluoro-DL-phenylalanine [31]. In these examples, the aim was to increase, rather than decrease, the compounds in question. The selection pressure used in the current study could be described as directionless, in that adaptation to chlorsulfuron does not guarantee reduced α-acetolactate production. One of the seven chlorsulfuron-adapted isolates had a slightly increased production of both VDKs, and the chlorsulfuron adaptation mechanism employed was presumably different to that in Isolate 8. Screening of chlorsulfuron-adapted strains for α-acetolactate production is clearly necessary to ensure that strains are suitable for application in brewing.

A positive outcome of the selection process was the apparent absence of any unwanted phenotypes in the evolved strain. Isolate 8 did not exhibit any loss in fermentation performance or growth, and volatile aroma compounds were largely unaffected. The specific nature of the change observed in Isolate 8 is likely due to the relatively mild conditions applied during mutagenesis and adaptation. Harsh selection conditions commonly result in strains that possess the phenotype selected for, but are otherwise unsuitable for, industrial application. Lee et al. [31] noticed, for example, that lager yeast adapted to 5,5,5-trifluoro DL-leucine for improved production of 3-methylbutylacetate had growth defects which reduced their potential for brewing. Likewise, unwanted flavor changes can occur. Attempts to increase the desirable 3-methylbutylacetate, can result in strains that have a similar increase in the corresponding higher alcohol 3-methybutanol, which imparts a less desirable taste to beverages [2]. The lack of unintended ‘side effects’ of the chlorsulfuron treatment may be due to the less acute toxicity of this compound compared to other sulfonylureas [8] and the sub-lethal concentrations used here for selection.

Gross chromosomal changes occurring during laboratory evolution included the loss of a copy of chromosome XV of the *S. cerevisiae* sub-genome and loss of one copy of a chimeric chromosome X [21] from both sub-genomes. It is not clear if these changes are in any way related to production of acetohydroxy acids, and the changes may rather be non-specific outcomes related to pre-selection mutagenesis or may somehow have conferred a faster growth rate to the cell line, thereby increasing the likelihood of selection. Change in chromosome copy number seems to be relatively common during adaptive laboratory evolution [42] and may represent an initial ‘quick-fix’ attempt at adaptation by the cell before the finer genetic adaptations are realized [47]. Of the gene-level changes observed, the most relevant is the proline to serine (P192S) mutation in *ILV2*. An early study showed that this same mutation confers resistance to sulfometuron methyl, a sulfonylurea compound related chemically to chlorsulfuron [46]. Subsequent work showed that this mutation is responsible for general resistance to sulfonylurea compounds [12]. The wild-type P192 proline interacts directly with the S-ring of sulfonylurea compounds, and the P192S mutation appears to desensitize yeast to these compounds [35]. While the reason for the enhanced resistance to sulfonylureas seems clear, the reason for the reduced production of α-acetolactate is less clear and further investigation of the kinetics of the acetohydroxyacid synthase with and without the mutation is necessary. Gjermansen et al. [19] also noted a lower level of diacetyl production in lager yeast adapted to sulfometuron methyl and it is possible that the same P192S mutation was responsible. The influence of the mutation on fermentation performance or other beer qualities was not reported in that instance. The likely role of the *ILV2* mutation in lowering diacetyl levels is also supported by the concomitant lowering of 2,3 pentanedione, a result which would be expected if a general loss of α-acetohydroxyacid synthase activity was responsible, rather than, say, a difference in uptake and utilization of valine.

The strategy undertaken here to control diacetyl levels is simple and cost effective, and does not negatively influence beer quality, requires no capital expenditure, no alteration of process conditions, no targeted metabolic engineering, no addition of exogenous nutrients or enzymes to the wort, and is achieved apparently with only a minor change to the yeast genome. As such, this approach is recommended for brewers seeking to reduce production times, energy expenditure and process costs, but without compromising beer quality.

## Electronic supplementary material

Below is the link to the electronic supplementary material.

Supplementary material 1 (DOCX 812 kb)

130 mutations observed in the variant strains Isolate 8 relative to A15 (XLSX 22 kb)
