# Supplementary material for: Diacetyl control during brewery fermentation via adaptive laboratory engineering of the lager yeast Saccharomyces pastorianus
Source: J Ind Microbiol Biotechnol. 2018 Oct 10;45(12):1103–12. doi: 10.1007/s10295-018-2087-4 (PMC6267509; doi:10.1007/s10295-018-2087-4)
Supplement: Supplementary file 1 — Supplementary material 1 (DOCX 812 kb) [file 10295_2018_2087_MOESM1_ESM.docx]

**Diacetyl control during brewery fermentation through adaptive laboratory engineering of the lager yeast *Saccharomyces pastorianus***

Brian Gibson^1^*, Virve Vidgren^1^, Gopal Peddinti^1^, Kristoffer Krogerus^1,2^

^1^ VTT Technical Research Centre of Finland Ltd, Tietotie 2, P.O. Box 1000, FI-02044 VTT, Espoo, Finland

^2^ Department of Biotechnology and Chemical Technology, Aalto University, School of Chemical Technology, Kemistintie 1, Aalto, P.O. Box 16100, FI-00076 Espoo, Finland

*Corresponding author. Tel: +358 207226603, Fax: +358 207227071, Email: brian.gibson@vtt.fi

#

**
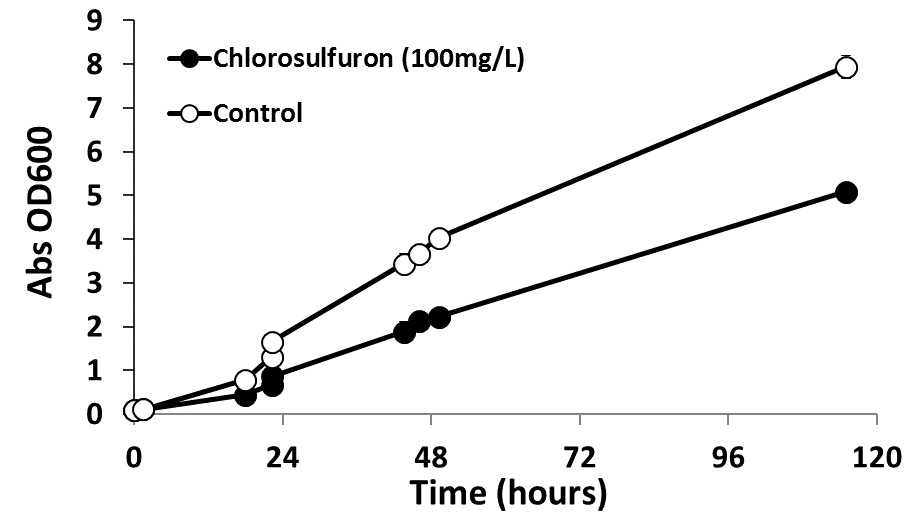
**

**Fig. S1.** Growth of the lager strain A15 in YNB medium containing maltose (4%, w/v) and supplemented with chlorsulfuron (100mg l^-1^). Values are means of two independent replicates and error bars where visible indicate the range about the mean.


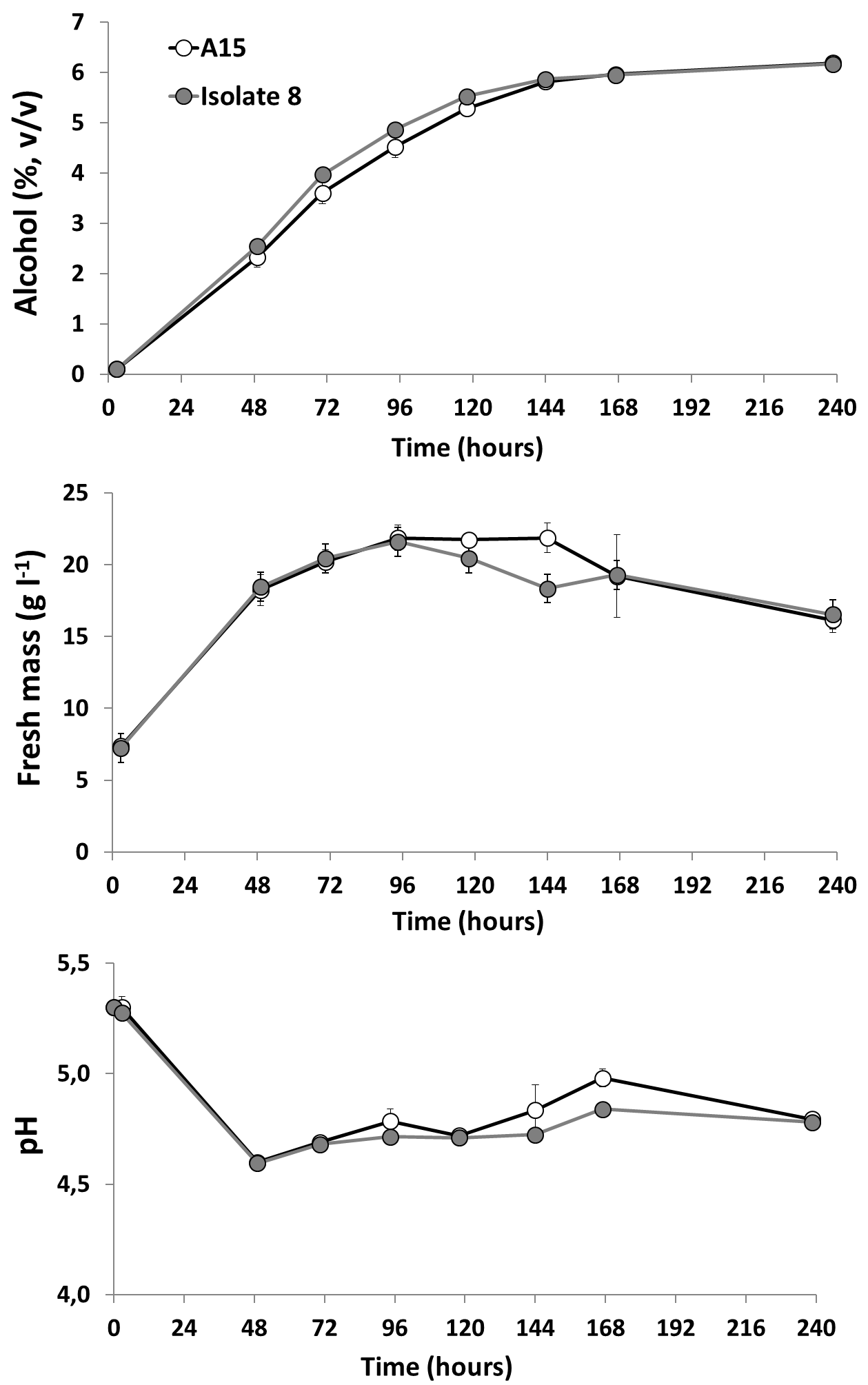


**Fig. S2.** Alcohol content, yeast mass in suspension and wort pH during fermentation of 15°P all-malt wort at 30L-scale with the lager strain A15 (open symbols) and an A15-derived, chlorsulfuron-adapted variant (Isolate 8; closed symbols). Values are means of two replicates and error bars where visible indicate the range about the mean.


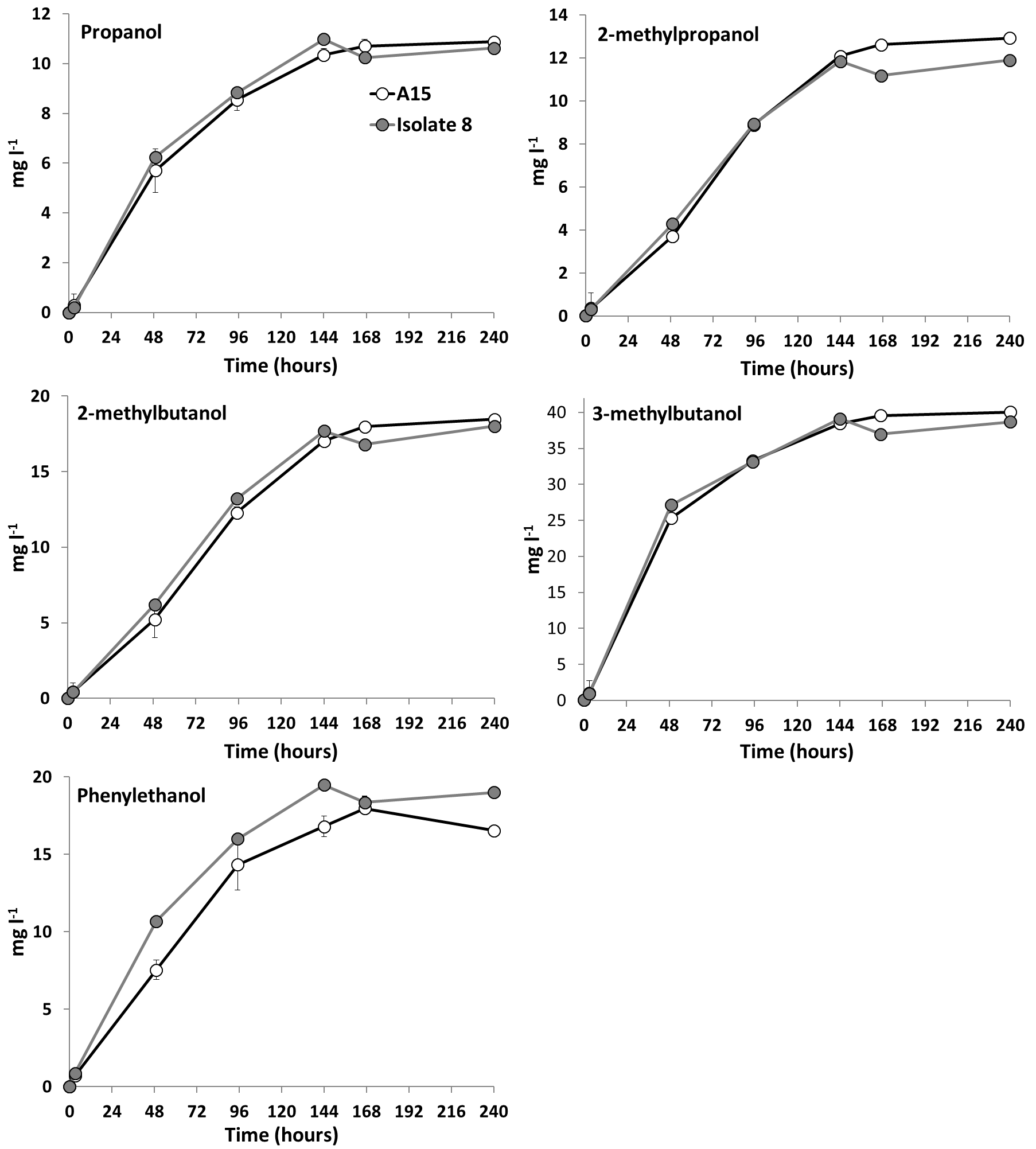


**Fig. S3.** Evolution of higher alcohols during fermentation of 15°P all-malt wort at 30L-scale with the lager strain A15 (open symbols) and an A15-derived, chlorsulfuron-adapted variant (Isolate 8; closed symbols). Values are means of two replicates and error bars where visible indicate the range about the mean.


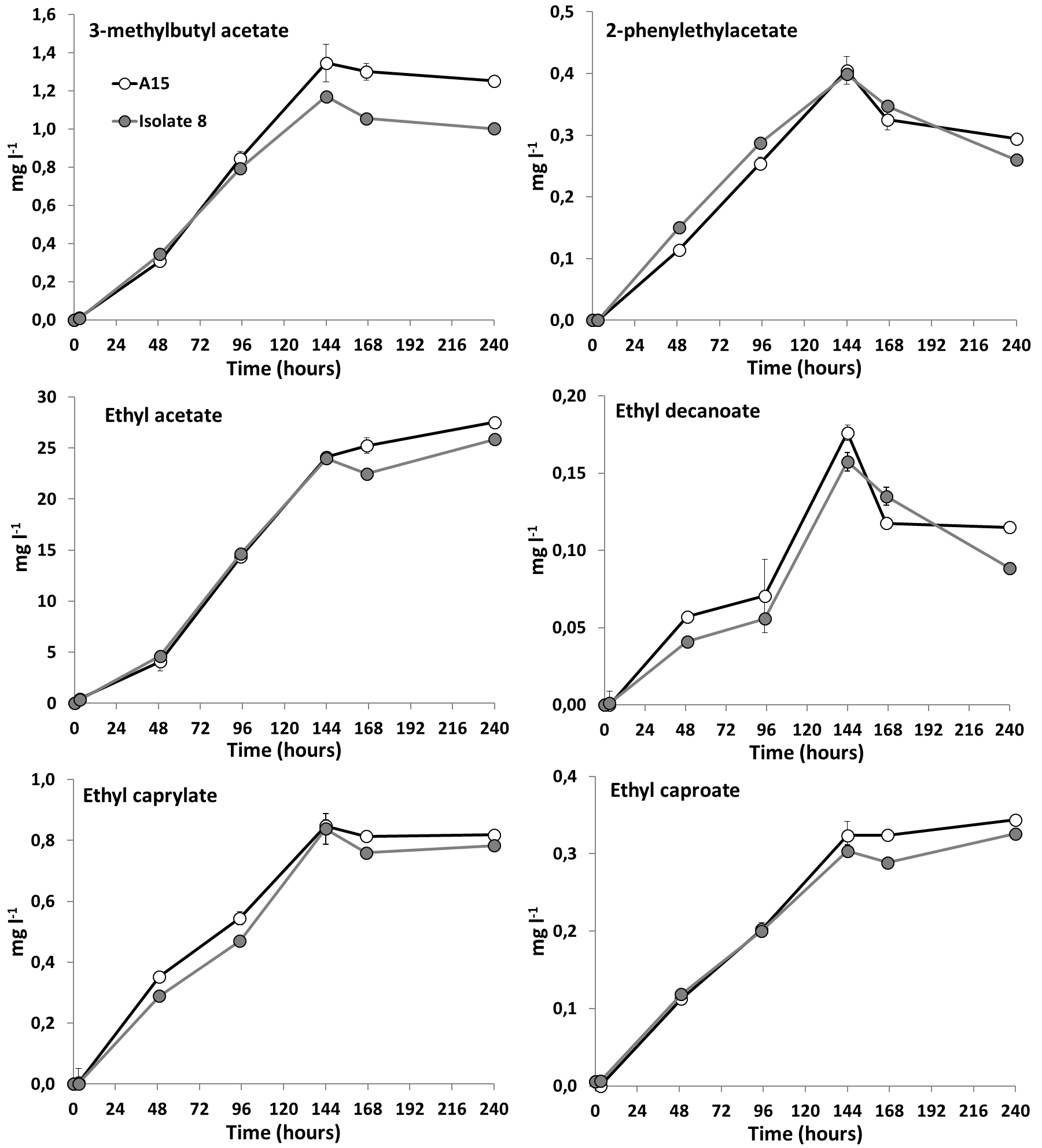


**Fig. S4.** Evolution of esters during fermentation of 15°P all-malt wort at 30L-scale with the lager strain A15 (open symbols) and an A15-derived, chlorsulfuron-adapted variant (Isolate 8; closed symbols). Values are means of two replicates and error bars where visible indicate the range about the mean.


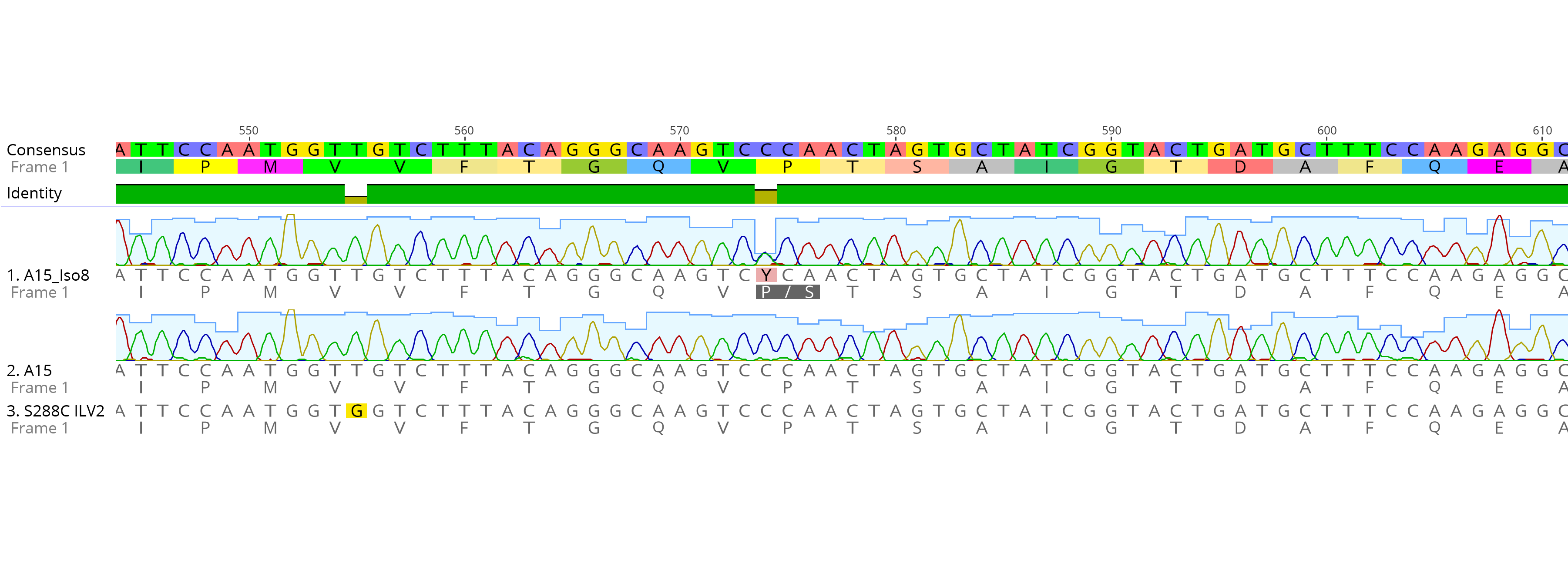
**Fig. S5.** Confirmation, via Sanger sequencing, of the heterozygous 574 C>T missense mutation in the *S. cerevisiae* allele of *ILV2* observed in the adapted variant of the lager yeast A15 (Isolate 8)
